# Phylodynamic reconstruction of O CATHAY topotype foot-and-mouth disease virus epidemics in the Philippines

**DOI:** 10.1186/s13567-014-0090-y

**Published:** 2014-08-24

**Authors:** Antonello Di Nardo, Nick J Knowles, Jemma Wadsworth, Daniel T Haydon, Donald P King

**Affiliations:** Institute of Biodiversity, Animal Health and Comparative Medicine, College of Medical, Veterinary and Life Sciences, University of Glasgow, Glasgow, G12 8QQ UK; The Pirbright Institute, Pirbright, Woking, Surrey GU24 0NF UK

## Abstract

Reconstructing the evolutionary history, demographic signal and dispersal processes from viral genome sequences contributes to our understanding of the epidemiological dynamics underlying epizootic events. In this study, a Bayesian phylogenetic framework was used to explore the phylodynamics and spatio-temporal dispersion of the O CATHAY topotype of foot-and-mouth disease virus (FMDV) that caused epidemics in the Philippines between 1994 and 2005. Sequences of the FMDV genome encoding the VP1 showed that the O CATHAY FMD epizootic in the Philippines resulted from a single introduction and was characterised by three main transmission hubs in Rizal, Bulacan and Manila Provinces. From a wider regional perspective, phylogenetic reconstruction of all available O CATHAY VP1 nucleotide sequences identified three distinct sub-lineages associated with country-based clusters originating in Hong Kong Special Administrative Region (SAR), the Philippines and Taiwan. The root of this phylogenetic tree was located in Hong Kong SAR, representing the most likely source for the introduction of this lineage into the Philippines and Taiwan. The reconstructed O CATHAY phylodynamics revealed three chronologically distinct evolutionary phases, culminating in a reduction in viral diversity over the final 10 years. The analysis suggests that viruses from the O CATHAY topotype have been continually maintained within swine industries close to Hong Kong SAR, following the extinction of virus lineages from the Philippines and the reduced number of FMD cases in Taiwan.

**Electronic supplementary material:**

The online version of this article (doi:10.1186/s13567-014-0090-y) contains supplementary material, which is available to authorized users.

## Introduction

Foot-and-mouth disease (FMD) is an economically devastating transboundary disease of cloven-hoofed domestic and wild ruminants, causing an acute and highly contagious vesicular disease which can develop into a persistent infection. The aetiological agent is FMD virus (FMDV), a single-stranded RNA virus belonging to the *Aphthovirus* genus, family *Picornaviridae*. FMDV is characterised by high genetic variability and exists as seven different serotypes named as O, A, C, Asia 1, Southern African Territories (SAT) 1, SAT 2, and SAT 3 [1]. As a consequence of their high mutation rate, FMDV lineages quickly diverge as they replicate and spread into new areas. Therefore, transmission of the virus through space and time directly defines the evolutionary patterns observed between related FMDV strains [2]. In addition to the accumulation of nucleotide substitutions through errors, large block of sequence changes can be mediated via recombination between different FMDV genomes, further expanding its evolutionary repertoire. In this context, FMDV populations often exhibit extensive genetic and antigenic heterogeneity at both the molecular and geographical level, driven by co-circulation of multiple lineages, heterogenic mixed host populations, extensive animal movements and trade patterns [3]. FMDV serotypes have evolved independently in different geographical regions to give rise to distinct genetic lineages, designated topotypes. Eleven topotypes have been defined for serotype O, based on phylogenetic relationships between available sequence data and a value of ~15% of nucleotide (nt) sequence difference in the VP1 coding region [4,5].

### The O CATHAY FMDV topotype

The first FMDV strain belonging to the O CATHAY topotype was isolated from Hong Kong SAR from pig samples collected during 1970 (HKN/21/70, GenBank accession no. AJ294911) and was characterised by a 93-102 nt deletion within the 3A coding region that is associated with the atypical porcinophilic phenotype of this FMDV lineage [6]. Subsequently, O CATHAY isolates have been confirmed in several Southeast and East Asian countries (including Malaysia, the Philippines, Taiwan, Thailand and Vietnam), although since 1970, the majority of field cases due to this topotype have been reported in Hong Kong SAR and China [7-9]. The O CATHAY FMD outbreak in Taiwan which began during 1997 resulted in the stamping-out of more than 4 million pigs and generated economic losses of over 6 billion US dollars [10]. Outside of Asia, viruses belonging to the O CATHAY topotype have been responsible for isolated FMD outbreaks that occurred in Europe in 1981 (Thalheim, Austria), 1982 (Wuppertal, Germany) and 1995 (Moscow, Russia). In the last ten years, O CATHAY FMDV strains causing epizootics have been collected in Hong Kong SAR on a yearly basis, where the last reported outbreak occurred during March 2014. However, FMD viruses belonging to the type O CATHAY topotype are sampled on a more sporadic basis from countries in Southeast Asia, and it is currently unclear where this topotype is maintained and/or how it is dispersed.

### FMDV in the Philippines

The introduction of FMD into the Philippines can be dated back to 1902 as a result of the importation of infected cattle from Hong Kong SAR to Manila. Following large epidemics reported in Sorsogon and Bukidnon Provinces in 1920, FMD became widespread in the entire Philippines. FMDV lineages belonging to serotypes A, O and C were identified in samples collected from outbreaks occurring in the Philippines during the period between 1954 and 2005. Major epidemics were caused by type O (from 1972 to 1991), type A (from 1975 to 1983) and type C (from 1976 to 1995) strains [11]. The O CATHAY topotype was first detected in August 1994 in a backyard piggery located in Rizal Province. More recently, this FMDV topotype has been the sole lineage responsible for epidemics in the Philippines until December 2005, when the last detected case was confirmed in Quezon Province. The majority of the cases due to O CATHAY were located on Luzon Island, from where FMD spread to 27 provinces. It has been estimated that wholesale market prices of both pork and even chicken in Central Luzon dropped significantly following the start of the epidemic in 1995, highlighting the economic impact of FMD across the entire supply chain [12]. Since June 2011, the Philippines have been officially declared as FMD-free (without vaccination).

This study explored the phylodynamics of these O CATHAY outbreaks reconstructed through molecular epidemiological analyses of VP1 coding sequences (*n* = 112) collected between 1994 and 2005. In addition, a wider picture of the O CATHAY topotype phylogenetics was determined from a larger database of currently available VP1 coding sequences (*n* = 322) to enable the characterisation of geographical movements of this FMDV lineage across historically affected countries of Southeast and East Asia.

## Materials and methods

### Sample database

This study accessed archived vesicular fluid and/or epithelium samples (*n* = 112) from the FAO World Reference Laboratory for FMD (WRLFMD) at The Pirbright Institute, United Kingdom, which had been stored at −20 °C in 0.04 M phosphate buffer (M25; disodium hydrogen phosphate, potassium dihydrogen phosphate, pH 7.5) and 50% (vol/vol) glycerol. This dataset represented clinical samples collected in the Philippines from 22 provinces in the period between 1994 and 2005 (Additional file 1). In addition, a further 210 VP1 coding region sequences and representing isolates collected from Austria, China, Germany, Hong Kong SAR, Malaysia, Russia, Taiwan, Thailand and Vietnam [8,13-18] were retrieved from both GenBank at NCBI [19] and the WRLFMD sequence archive and, then, integrated with the Philippines collection to comprise a total dataset of 322 VP1 coding sequences (Additional file 2) These VP1 coding region sequences have been submitted to GenBank as have been assigned the following accession numbers: KM243030-KM243172.

### Viral RNA detection and sequencing

Clinical samples were processed in order to obtain the FMDV VP1 coding sequences (639 nt length, ~8% of the full genome length). Viral RNA for each sample was extracted from virus suspensions using the RNeasy® Mini Kit (QIAGEN® Ltd., UK), according to the manufacturer’s protocol. One-step RT-PCR to amplify the VP1 region of FMDV was carried out as previously described [20]. Primers used for the RT-PCR step were O-1C244F and O-1C272F for the forward, and EUR-2B52R for the reverse orientations (Table 1). PCR products were cleaned up using the Illustra GFX™ PCR DNA and Gel Band Purification Kit (GE Healthcare Ltd., UK), and were then cycle-sequenced using the BigDye® Terminator v3.1 Cycle Sequencing Kit (Applied Biosystems, UK). A set of reverse and forward primers was employed to ensure the complete coverage of the VP1 coding region (Table 1). Sequencing reactions were analysed using the ABI 3730 DNA Analyzer (Applied Biosystems, USA). Raw data files were assembled into a contig and edited using SeqMan Pro™ 11.2 (DNASTAR, Inc.), then aligned using Clustal Omega 1.2.0 [21].

**Table 1:** **Oligonucleotide primers used for either RT-PCR or cycle sequencing of the VP1 region from the FMDV isolates**

**Primer designation**	**Primer sequence (5΄ to 3΄)**	**Start - end**
*Reverse primers*		
NK72	GAAGGGCCCAGGGTTGGACTC	3558 – 3578
EUR-2B52R	GACATGTCCTCCTGCATCTGGTTGAT	3624 – 3649
O-1D487gR	TAATGGCACCRAAGTTGAA	3372 – 3390
O-1D628R	GTTGGGTTGGTGGTGTTGT	3181 – 3199
*Forward primers*		
O-1C244F	GCAGCAAAACACATGTCAAACACCTT	2469 – 2494
O-1C272F	TBGCRGGNCTYGCCCAGTACTAC	2497 – 2519
O-1C283F	GCCCAGTACTACACACAGTACAG	2508 – 2530
O-1D296F	ACAACACCACCAACCCAAC	3181 – 3199
O-1C499F	TACGCGTACACCGCGTC	2724 – 2740
O-1C605hF	TGGCCAGTGCCGGTAAGGACTTTGAC	2830 – 2855
O-1C605nF	TGGCTAGTGCTGGCAAAGACTTTGAC	2830 – 2855

Start and end locations have been mapped against the Kaufbeuren/FRG/66 type O FMDV isolate (GenBank accession no. X00871) [22].

### Phylogenetic analysis

Before performing the phylogenetic reconstruction, jModelTest 2.1.4 analysis [23,24] was undertaken to determine the best fitting nucleotide substitution model using the Bayesian Information Criterion (BIC) [25]. Statistical parsimony [26] was used for reconstructing the genealogical networks as implemented in the TCS 1.21 program [27]. The network generated was then edited and plotted in yEd Graph Editor 3.12.

A Bayesian analysis framework was employed for phylogenetic and demographic inferences using a Markov chain Monte Carlo (MCMC) method implemented in the BEAST 1.8.0 package [28]. The analysis was performed using the Hasegawa-Kishino-Yano substitution model plus gamma-distributed rates (HKY85 + Γ4), and the relaxed uncorrelated lognormal molecular clock model [29,30]. Demographic reconstruction was employed using the Bayesian skyline model [31]. Spatial patterns of FMDV dispersal were estimated through a probabilistic discrete asymmetric diffusion model using a continuous-time Markov chain process, adopting a Bayesian stochastic search variable selection (BSSVS) procedure to select among all possible migration pathways [32]. Nonzero rates of virus movement between countries were judged to be supported when the associated Bayes factor (BF) exceeded 3. The MCMCs were run for 150 million iterations, sub-sampling every 15 000 states. Convergence of the chain was assessed using Tracer 1.5 removing the initial 10% of the chain as burn-in. The maximum clade credibility (MCC) tree was summarised using TreeAnnotator 1.8.0 and constructed using FigTree 1.4.0. Phylogeographic maps were constructed using ArcGIS 10.2.1 (Environmental Systems Research Institute, Inc.).

### Statistical analysis

The epidemic curve was constructed using the Handistatus II data for the Philippines retrieved from the OIE website [33]. Statistical computations were performed in R 3.0.3 [34] and graphs were plotted using the ggplot2 package for R [35], whereas complex vector images were rendered using Inkscape 0.48.4. To determine the potential extent of recombination in the genetic structuring of the virus population, ratios of per-site recombination rate to the per-site mutation rate (*r*) were estimated using LAMARC 2.1.9 [36].

## Results

### O CATHAY FMDV country based phylodynamics: the Philippines

A FASTA search [37] of all publically available VP1 coding sequences was completed to identify a candidate for the most likely common ancestor for the Philippines lineage: the closest match was identified as a sequence from Hong Kong SAR with 99.2% nt identity (HKN/12/91, GenBank accession no. AJ294921).

**Figure 1:**
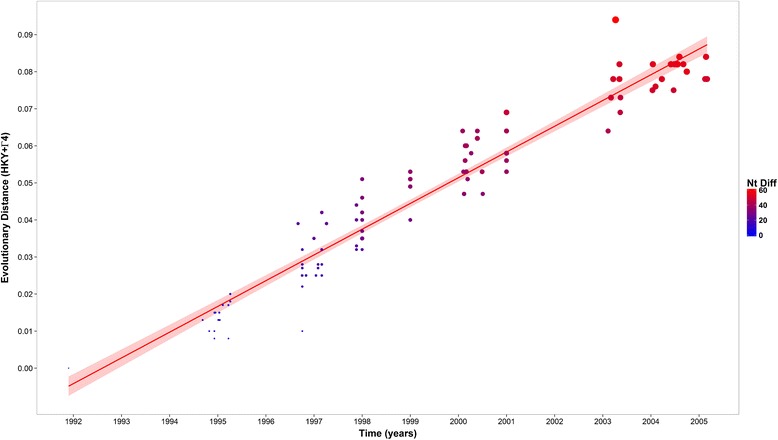
**Accumulation of nucleotide differences calculated from the putative root (HKN/12/91 isolate) for the Philippines database with time expressed in years.** Size of the points increases with increased number of nt substitutions. Shaded area represents 95% confidence intervals for the fitted line.

The observed evolutionary distances and total nt changes calculated from the root (HKN/12/91) increased linearly with time (R^2^ = 0.932; F_1, 111_ = 1528, *p* < 0.001) (Figure 1). The number of nt substitutions in the VP1 coding sequences between the first O CATHAY isolate collected in the Philippines in 1994 and the last reported outbreak in 2005 was estimated to be 58, although the maximum number of nt substitutions was reported for the PHI/17/2003 isolate as 69 (maximum genetic distance 0.12 base substitution per site). No indels were found within the entire alignment. In addition, variability in the number of nt changes in samples collected within the same time window (year) was observed. Average genetic divergences among year groups were estimated to be higher for 2000, 2001 and 2003, which deviate from the average value of 0.023 ± 0.008 base substitutions per site per year (Table 2). Geographic distance was found to be significantly correlated with genetic distance (F_1, 84_ = 15.92, *p* < 0.001). A recombination rate (*r*) of 8.76 × 10^−8^ per site per generation (site/generation) was estimated for the Philippines indicative of an exceedingly low rate of recombination relative to mutation.

**Table 2:** **Genetic, time and geographical pairwise distances (with corresponding standard deviation values) calculated for the within-year Philippines O CATHAY FMDV isolates groups and for each of the country based data from the earliest samples collected within the specific group**

**Data**	**No of samples**	**Genetic distance**	**Time distance**	**Geo distance**
*Philippines*				
1994	7	0.010 ± 0.002	0.23 ± 0.05	-
1995	8	0.013 ± 0.006	0.63 ± 0.77	124.72 ± 95.81
1996	9	0.020 ± 0.004	0.10 ± 0.03	235.66 ± 252.21
1997	14	0.016 ± 0.013	0.38 ± 0.38	119.65 ± 117.12
1998	23	0.011 ± 0.009	-	51.62 ± 42.08
1999	5	0.011 ± 0.002	-	146.83 ± 222.79
2000	16	0.054 ± 0.021	0.16 ± 0.13	296.55 ± 130.74
2001	7	0.057 ± 0.008	-	14.22 ± 4.28
2003	8	0.051 ± 0.013	0.19 ± 0.08	344.45 ± 26.98
2004	12	0.010 ± 0.004	0.41 ± 0.23	322.32 ± 38.83
2005	3	0.005 ± 0.005	0.03 ± 0.01	12.48 ± 17.65
*Global*				
China	6	0.148 ± 0.081	31.93 ± 19.15	-
Hong Kong	138	0.157 ± 0.022	32.95 ± 7.24	-
Philippines	112	0.047 ± 0.022	4.59 ± 3.50	-
Taiwan	46	0.015 ± 0.025	1.38 ± 3.01	-
Vietnam	13	0.104 ± 0.016	7.84 ± 1.65	-

Genetic distances were estimated by the Hasegawa-Kishino-Yano substitution model plus gamma-distributed rates (HKY85 + Γ4), whereas geographic distance were calculated using the Haversine formula [38]. Genetic distance is expressed in base substitution per site, time distance is defined in years, whilst geographical distance is measured in kilometres.

**Figure 2:**
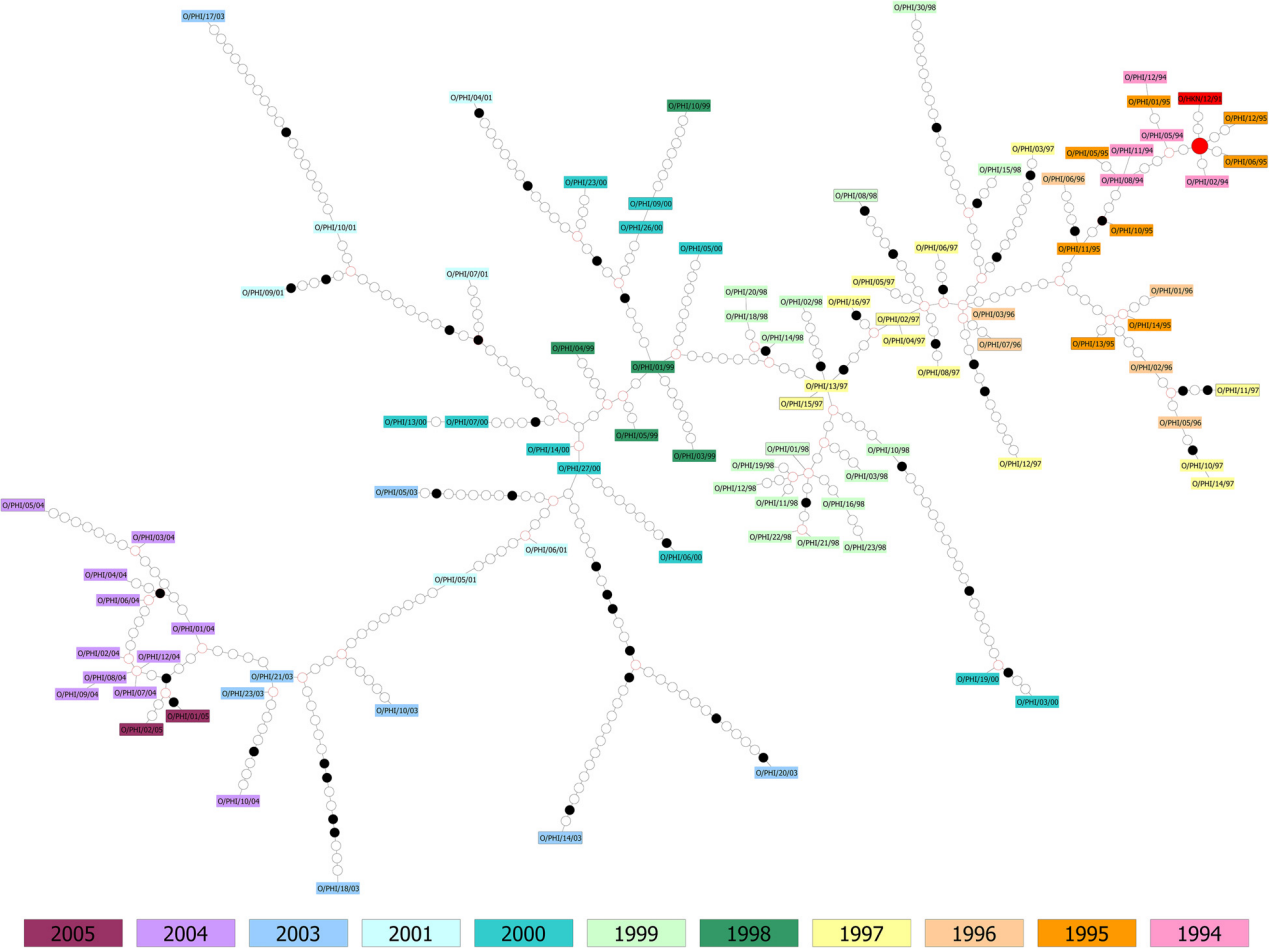
**Network extracted from the statistical parsimony analysis performed in TCS for the Philippines isolates (** ***n*** **= 112).** VP1 sequences are designated with their WRLFMD number and coloured by year of collection, where the outlier (HKN/12/91) is defined with a red box. The MRCA for the Philippines O CATHAY FMDV taxon is highlighted in a red ellipse. Black dots specify non-synonymous substitutions, whereas white dots represent synonymous substitutions. The year codes in the virus isolate labels have been abbreviated to the last two digits.

**Figure 3:**
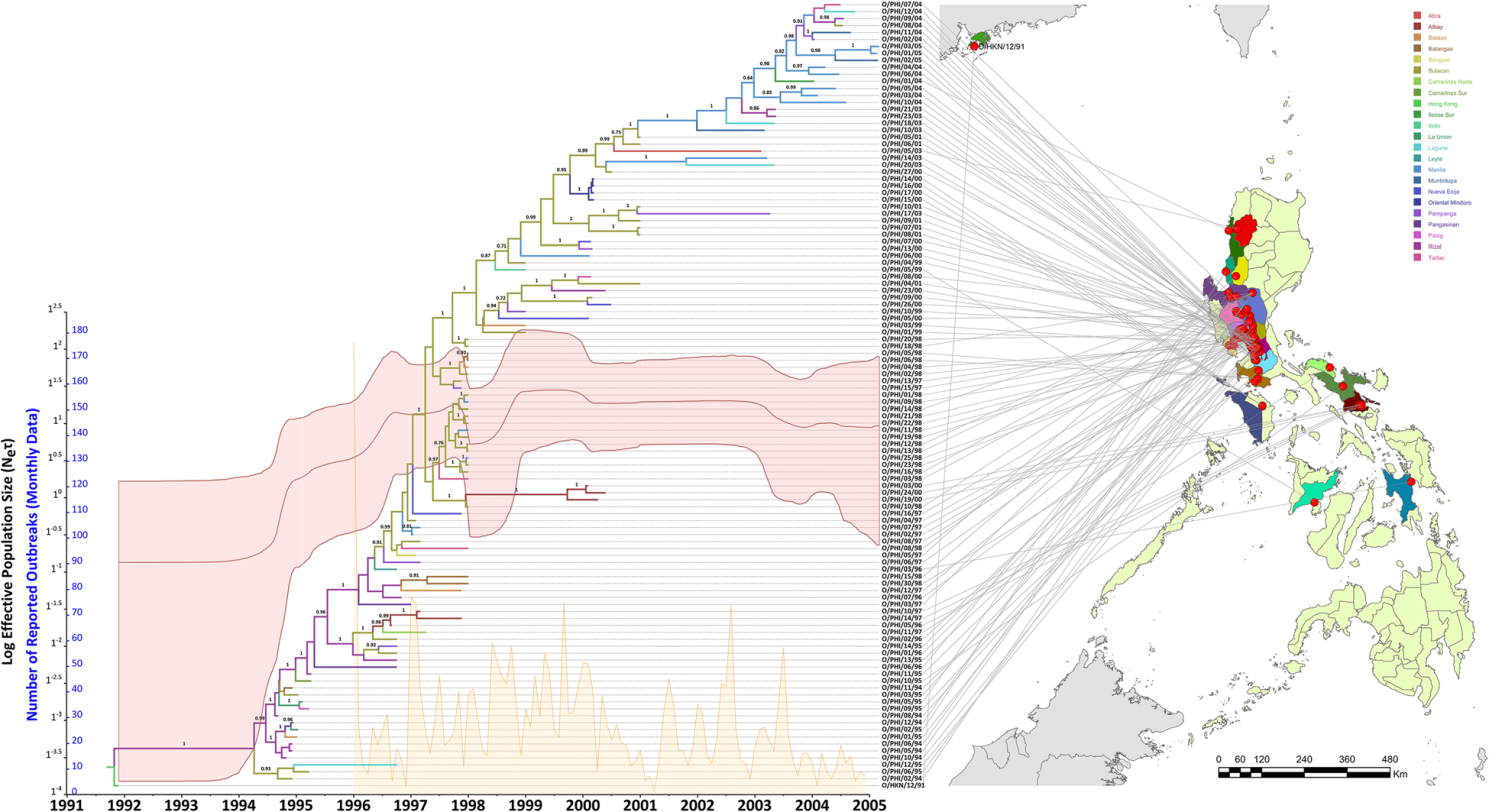
**Phylodynamic reconstruction of the O CATHAY FMDV epidemics in the Philippines.** Maximum clade credibility tree branches are coloured according to the most likely transmission source as reconstructed from the discrete states model. Nodes with a posterior probability value ≥ 0.7 are shown. FMDV demography is expressed by log Effective Population Size (N_e_τ) as estimated from the Bayesian skyline plot along with the monthly epidemic curve reconstructed from the data retrieved from the OIE Handistatus II database.

As estimated by the statistical parsimony network analysis, the most recent common ancestor (MRCA) of the Philippines O CATHAY taxon was identified as an unsampled virus 3 nt different from HKN/12/91 and 1-3 nt different from the earliest Philippines isolates collected between late 1994 and the start of 1995 (Figure 2). The diameter of the parsimony network between the MRCA and the most divergent FMDV isolate collected in 2004 (PHI/5/2004) was estimated to be 86 nt substitutions, of which 83 (96.51%) were synonymous and 3 (3.49%) non-synonymous. The average of number of nt substitutions incurred per year (nt/yr) of any isolate from its closest sampled ancestor was estimated to be 9.9 ± 4.8, comprising an average of 8.8 ± 4.2 synonymous and 1.0 ± 0.9 non-synonymous changes, indicative of an average rate of change for VP1 sequences in the Philippines of approximately 1.5% per year. The average number of changes for each isolate was 4.0 ± 2.3 nt/yr, of which 3.4 ± 2.1 and 0.6 ± 0.5 were synonymous and non-synonymous substitutions, respectively. Most sequences clustered according to time across the network, although FMDV isolates collected in 2000 were assigned within three separate genetic lineages, resulting in three evolutionary pathways one of which was a dead-end. In addition, for some links more recently collected viruses were assigned earlier in time on the network. The case of PHI/12/94 which was found to be a descendant of PHI/1/95 can in part be explained by the short time distance which separates these two isolates (32 days) and it might be that both strains (or their ancestors) were co-circulating at time of sampling. The reconstructed phylogeny further defined these two viruses as being closely related (genetic distance of 0.002 base substitutions per site). Conversely, samples collected in March (PHI/9/2000) and June 2000 (PHI/26/2000) were determined to be the source of a virus collected in 1999 (PHI/10/99), although the 2000 isolates were direct descendants of a virus detected in January 1999 (PHI/1/99). Looking in detail at this case, the phylogeny found descent of PHI/10/99, PHI/9/2000 and PHI/26/2000 from the same common ancestor. These samples were collected from the same region (Central Luzon) within an area of ~40 km of radius, potentially explaining the inconsistent result provided by the TCS analysis to have arisen from sampling bias. The discrete states analysis resolved the relationship of the PHI/10/99, PHI/9/2000 and PHI/26/2000 isolates rooting those from a common ancestor that descends in turn from an unsampled virus source both seeded from Bulacan Province, which includes the PHI/1/99 sample (Figure 3).

The molecular clock for the O CATHAY Philippines lineage was estimated to be 1.25 × 10^−2^ nt/site/yr (95%HPD 9.47 × 10^−3^ to 1.57 × 10^−2^) with a standard deviation of 0.70 (95%HPD 0.49 to 0.91). No evidence of autocorrelation of rates in the reconstructed phylogeny was provided by the covariance value of 2.65 × 10^−3^. The introduction date, the time of the MRCA (TMRCA), of the type O CATHAY topotype FMDV lineage into the Philippines was calculated to be the 30^th^ of March 1994 (95%HPD 07/08/1993 to 08/08/1994, a time interval which included the date of the first officially reported case).

The reconstructed FMDV population dynamics from the skyline plot (Figure 3) describes a demographic history characterised by three phases. In the first phase, after an initial exponential increase from mid-1994 until late 1997 at a rate that decreased from late 1996, a sudden and short period of decline was observed, resulting in a population bottleneck. Since genetic bottlenecks correspond to significant reductions in population size, these changes in the O CATHAY population dynamics in the Philippines probably link to the launch of an extensive control plan in 1996 that was successful in limiting the further spread of FMD and thereby reducing the number of outbreaks [11]. However, during 1999 a new FMD outbreak occurred within an already declared FMD-free zone, the Panay region. Therefore in the second phase, the skyline trajectory recorded a second rapidly increasing viral population size starting in mid-1998 and lasting up to the first months of 1999, which resulted in a diversification of viral lineages. In the third phase, the viral population size reached a plateau until late 2002, when further control policies resulted in a steady decline in FMD prevalence until eradication.

The epidemic curve drawn from the field epidemiological data from the OIE for the period 1995-2005 [33] described an oscillatory trend in the number of FMD outbreaks reported in the Philippines, with times of high epidemic peaks interleaved by low-level FMD circulation. The frequency of these oscillations was higher between 1997 and the beginning of 2000 (a monthly average of 37.9 FMD outbreaks), after which the number of FMD outbreaks started to decline following periods of low reporting (with a monthly average of 19.8 FMD outbreaks). However, the reported epidemic trend did not overlap with the skyline plot trajectory, although the epidemic window from mid-2000 to 2005 characterised by a reduced number of outbreaks could be evinced by the plateauing and subsequent decrease in the genetic diversity of the skyline plot. It should be noted that although more than 300 outbreaks were officially reported through OIE during 2002, no clinical samples (and thus genetic information) were collected within that time window.

According to the results obtained by the discrete states phylogeography analysis, the root of the Philippines taxon was found to be from Rizal Province, consistent with the location of the first officially reported cases of O CATHAY topotype in the Philippines during August 1994 (Figure 3). Three main epidemic hubs could be identified from the analysis: the first from the beginning of the epizootic up to mid-1996, where outbreaks were found to be seeded from Rizal Province; the second lasting until 2001, where Bulacan Province was estimated to be the main source of FMD spread; and lastly, Manila Province as the last epidemic hub. The movement transitions between the three main epidemic hubs were supported by Bayes factor values of > 24 [posterior probability (pk) = 1.0] for movements from Rizal to Bulacan and from Bulacan to Manila, respectively.

### Global and regional phylodynamics of O CATHAY topotype FMDV

The molecular clock rate for all the O CATHAY topotype VP1 data was estimated to be 1.06 × 10^−2^ nt/site/yr (95%HPD 8.99 × 10^−3^ to 1.23 × 10^−2^), with a standard deviation of 0.81 (95% HPD 0.67 to 0.94). This value was comparable with the molecular clock rate reported for the Philippine isolates only. The MRCA for the O CATHAY topotype was estimated to have been present between 1955 and 1960. The *r* recombination parameter returned a value of 8.3 × 10^−9^ site/generation indicating a very low influence of recombination relative to mutation.

**Figure 4:**
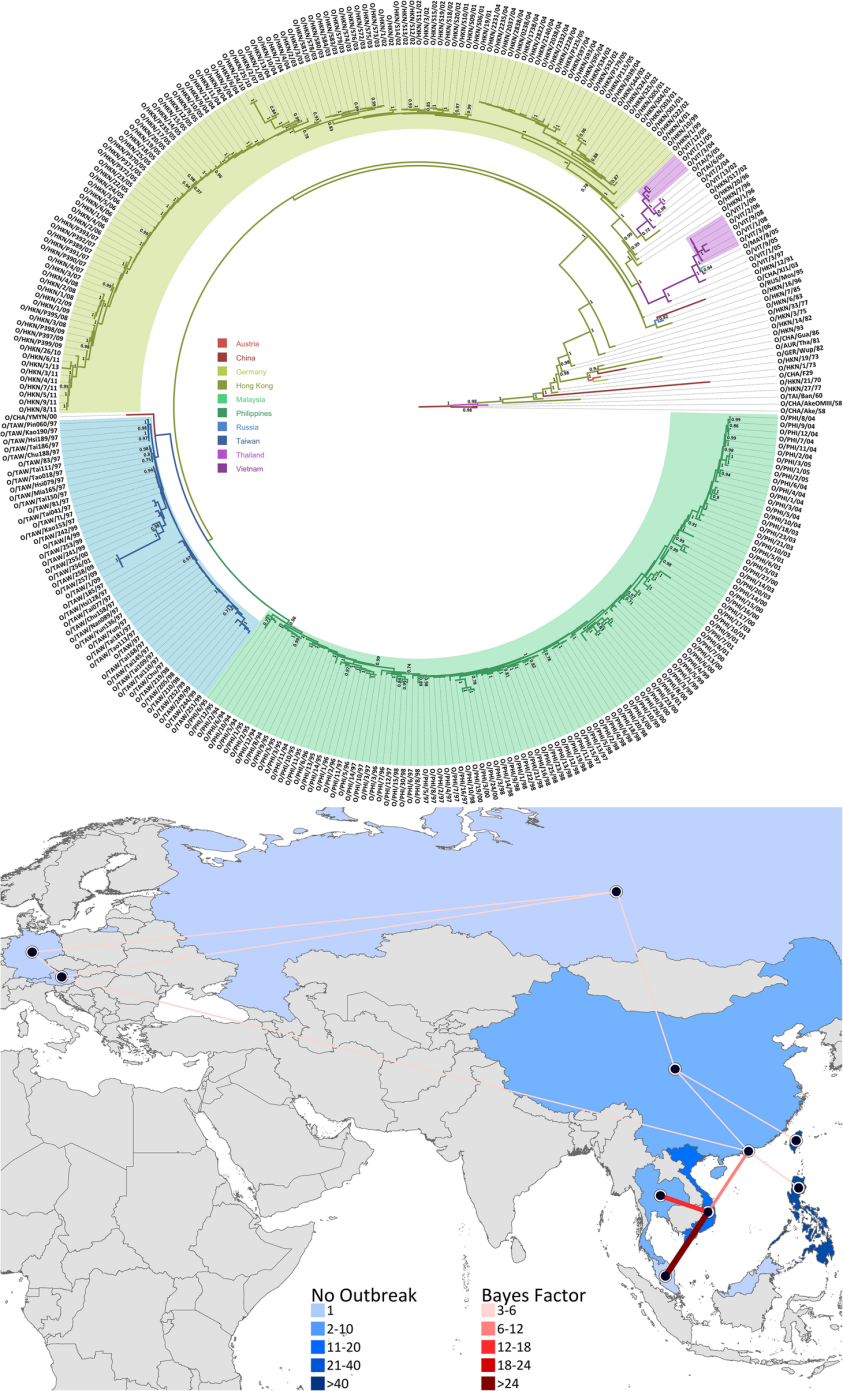
**Maximum clade credibility tree for all the O CATHAY FMDV isolates sequenced (** ***n*** **= 322).** Nodes with a posterior probability value ≥ 0.7 are shown. Branches are coloured according to the most probable country of the node from which they descended as estimated from the discrete state phylogeographic Bayesian model. Geographical links between countries identified by the BSSVS analysis are coloured by the corresponding BF value. The year codes in virus isolate labels have been abbreviated to the last two digits. The geographical locations are defined with the country centroid.

Three distinct sub-lineages were identified by the wider phylogenetic reconstruction that included the full database of O CATHAY VP1 coding sequences, which were clustered on a country level basis (Figure 4). The FMDV strains circulating in the Philippines were found to have descended from a common ancestor that was shared with the Taiwanese isolates, in line with what was proposed to be the source of introduction of the O CATHAY virus into the Philippines in 1994 [8]. In turn, the Taiwanese cluster descended from an unsampled virus closely related to a FMDV isolate collected from China in 2000. The Hong Kong SAR isolates were defined in a separate phylogenetic cluster along with FMDV samples collected from countries in mainland Southeast Asia (Malaysia, Thailand and Vietnam). This finding is in contrast to that previously reported [18], which designated the Taiwanese lineages descending from a common ancestor with the Hong Kong SAR isolates, and identified the Philippines lineages as a distinct phylogenetic cluster. Hui and Leung [18] inferred the phylogenetic relationship employing a Neighbor-joining method; nevertheless, estimating the phylogeny using a maximum-likelihood method [39] did not alter the shape of the reconstructed phylogeny (data not shown). The three phylogenetic clusters shared a common ancestor related to a FMDV strain collected in Hong Kong SAR in 1991 (HKN/12/91), which was in turn a descendent from other Hong Kong SAR isolates related to more recent samples obtained from Russia (1995), Hong Kong SAR (1996) and China (2003). FMDV isolates collected from countries of mainland Southeast Asia were phylogenetically grouped into two distinct clusters: the first (MRCA dated 1997) including the first O CATHAY virus isolate from Vietnam in 1997 from which viruses were collected in 2005-06 and 2008, and the only isolate from Malaysia (2005) was sourced; the second (MRCA dated 1998) associated with a later introduction of an O CATHAY strain in Vietnam in 2002, from which viruses isolated in 2004-05, and FMDV sequences from Thailand (2005) were related. The FMDV ancestor of the first mainland Southeast Asia sublineage was dated circa mid-1993, directly descending from the oldest MRCA of the Hong Kong SAR cluster, whereas the second sublineage was circulating in late 1998 and closely related to a virus collected in Hong Kong SAR in 2002. This phylogenetic picture supports two potential introductions of the O CATHAY FMDV lineage into Vietnam from Hong Kong SAR.

The MRCA shared between the Philippines and Taiwanese phylogenetic clusters was estimated to have been circulating in 1993 (95%HPD 1992 to 1994), whereas the origin of the MRCA for the more recent O CATHAY FMD epidemics in the Southeast and East Asia regions was dated 1991 (95%HPD 1990 to 1992). No other virus introduction or escape was ascribed to the Philippines O CATHAY FMD epidemic history, suggesting the Philippines sub-lineage to be monophyletic. In contrast, Hui and Leung [18] described two different FMDV introductions into the Philippines, assigning the PHI/5/95 isolates within the phylogenetic cluster which includes the Taiwanese isolates. However, the tree node that governed this inclusion had a bootstrap value of < 70, suggesting uncertainty in the assignment of these descendants.

**Figure 5:**
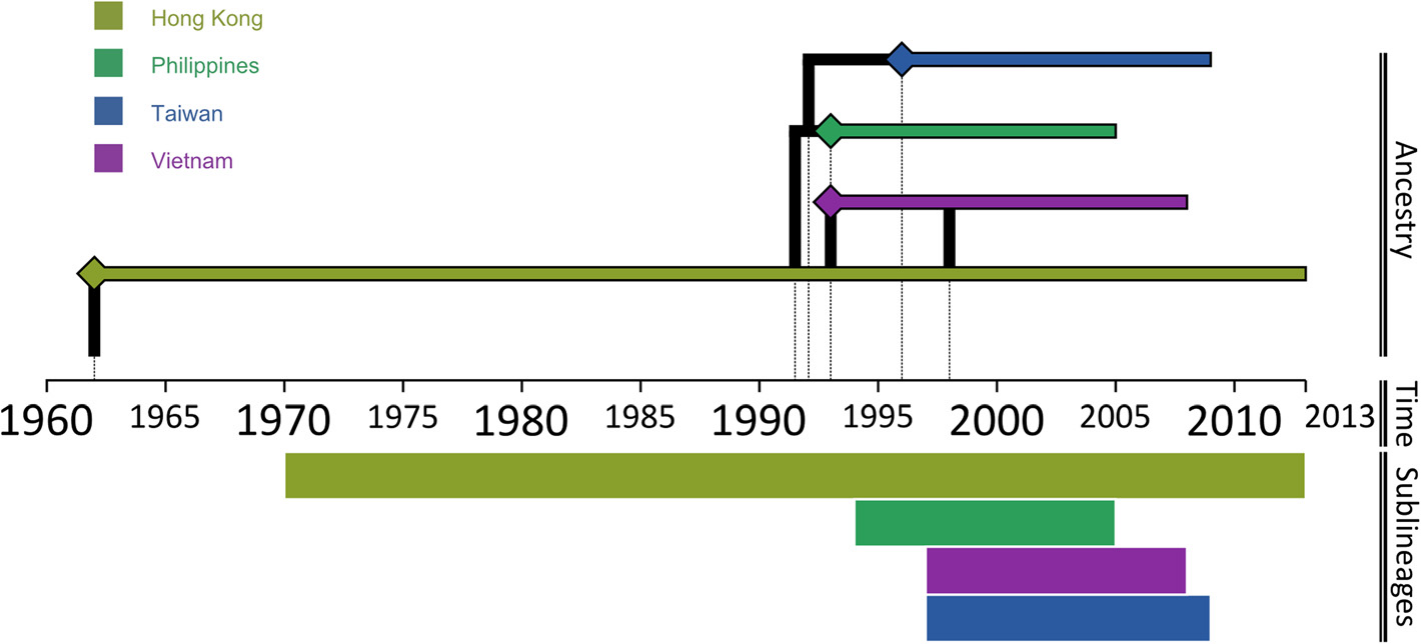
**Chronological evolutionary trend and transmission ancestry of the O CATHAY FMDV topotype in Southeast Asia.** Spatio-temporal reconstruction of the historical movements of the FMDV type O CATHAY lineage across Asia.

As estimated by the discrete phylogeography model, the root of the entire phylogenetic tree was reported to be in Hong Kong SAR and, therefore, representing a likely source for the introduction of the O CATHAY lineage into the Philippines and Taiwan. This is confirmed by the estimated BSSVS parameters, for which China and Hong Kong SAR were assessed as the main hubs of FMDV spread between countries (Figure 4): China was found to be the source for Hong Kong SAR (BF = 5.6, pk = 0.60), Taiwan (BF = 5.07, pk = 0.58) and Russia (BF = 4.33, pk = 0.54), whilst Hong Kong SAR was identified as the source of FMD transmission to Vietnam (BF = 6.75, pk = 0.65) and the Philippines (BF = 3.16, pk = 0.46). The link found between China and Russia reinforces the hypothesis that Chinese pork shipments were responsible for the introduction of the O CATHAY lineage into Moscow, Russia during 1995 [6]. Vietnam was estimated as a recipient of viruses moving from Malaysia (BF = 23.25, pk = 0.86), Thailand (BF = 12.55, pk = 0.77) and Hong Kong SAR (BF = 6.75, pk = 0.65). The most likely routes of introduction of the FMDV O CATHAY lineage into Europe were identified to be from Russia to Austria (BF = 3.92, pk = 0.32), although Russia to Germany (BF = 3.51, pk = 0.30) and Hong Kong SAR to Austria (BF = 3.14, pk = 0.27) were also considered as possible movement routes. The virus movement within Europe has been identified from Austria to Germany (BF = 5.15, pk = 0.58). Thus supported by the Bayesian phylogenetic and BSSVS analyses, the historical movement of the FMDV type O CATHAY lineage across Asia might be temporally and spatially reconstructed as represented in Figure 5.

**Figure 6:**
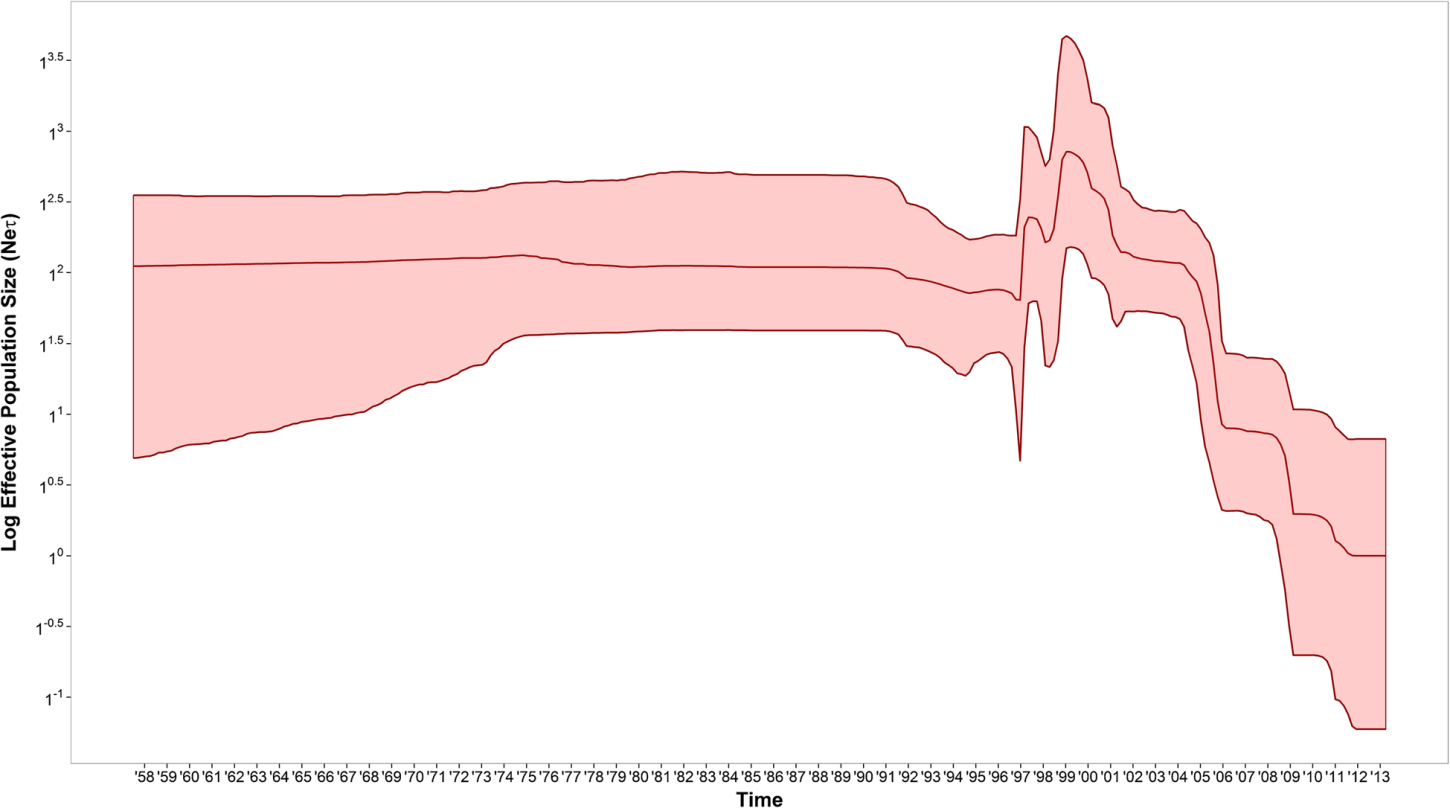
**Skyline plot of log effective population size (N**_**e**_ **τ) against time in years estimated from the full O CATHAY FMDV database.** Light red ribbon defines the 95% high posterior density interval area.

The historical phylodynamics of the FMDV O CATHAY lineage, as reconstructed by the skyline model using the full currently available VP1 coding sequences database (Figure 6), underwent three distinct and chronologically consequent evolutionary stages. In the initial stage, the genetic diversity was roughly constant until 1997, after which there were two increasing phases within a period of 3 years from 1997 to 2000, with the highest peak in 1999. The last stage is characterised by four sequential declining phases, with a rapid sharp drop between 2004 and 2006. This triphasic phylodynamic feature might be associated with an oscillatory tendency of FMDV genetic diversity driven by a first expansion phase due to the introduction of the virus into Taiwan and Vietnam and the trigger of the Philippines epidemic, and a later contraction phase following steps taken to eradicate the disease from the Philippines and the decrease in the number of outbreaks reported from Taiwan, characterised by the period between 2001 and 2009 when few cases were reported. This assumes that the FMDV type O CATHAY topotype has been maintained constantly within the Hong Kong SAR livestock system.

## Discussion

The evolutionary dynamics of the O CATHAY topotype of FMDV have been analysed allowing the transmission dynamics to be reconstructed across countries in Southeast Asia that have been impacted by this lineage. The O CATHAY FMDV strains isolated from outbreaks reported in Hong Kong, Taiwan and Philippines were defined as belonging to three different sublineages, which were related by a shared common ancestry to an unsampled FMDV strain sourced from Hong Kong SAR. The O CATHAY FMD epizootic in the Philippines resulted from a single introduction and was characterised by three main transmission hubs in Rizal, Bulacan and Manila. Although the evolutionary dynamics of the O CATHAY FMDV lineage were described by three phases from the skyline reconstruction, this was not entirely consistent with the monthly epidemic curve (Figure 3). This could be either due to a spatio-temporal bias in the genetic information analysed or in the incompleteness of the outbreak reporting database used, or both.

The phylodynamics of FMDV reconstructed from the FMDV type O CATHAY VP1 coding sequences indicates a marked reduction in viral diversity in the last 10 years, corresponding to the eradication of FMD in the Philippines and the more limited disease events experienced in Taiwan. Furthermore, the introduction of the FMDV type O Southeast Asia (SEA) topotype Mya-98 lineage into Hong Kong SAR during 2010 could have reduced the genetic diversity within O CATHAY lineages through direct competition with available hosts, as well as the presence of cross-protective antibodies in convalescent animals. These findings indicate that the O CATHAY topotype is maintained in the Hong Kong SAR ecosystem and sporadically spread from there to other Southeast Asian countries, as would be the case for the Philippines in 1994 and Vietnam in 1997. However, few O CATHAY FMDV strains have been reported from mainland China, which has the largest swine production industry in the world (representing over 51% of the world’s pig population). These few isolates were collected in 1986, 2000, 2001 and 2003, therefore sampling bias or underreporting of epidemic events occurring in China would likely have an impact on assessing the geographical movements of the FMDV type O CATHAY topotype. It is, nevertheless, clear from the analysis that a transmission link exists between China and Hong Kong SAR, thus indicating a historically southward movement of the O CATHAY FMDV lineage.

The molecular clock estimated here for the O CATHAY topotype is at the high end of evolutionary rate estimates for FMDV. Previously estimates reported an average evolutionary rate across all FMDV serotype of 2.48 × 10^−3^ nt/site/yr [40], while rates of 3.14 × 10^−3^, 1.3 × 10^−3^ and 4.8 × 10^−3^ nt/site/yr were reported for serotype O [40-42]. In addition, lineage-based FMDV molecular clock rates of 2.8 × 10^−3^, 6.65 × 10^−3^, 7.81 × 10^−3^ and 2.7 × 10^−3^ nt/site/yr were previously estimated for the O-PanAsia lineage in India, O-PanAsia-2 sublineage in Pakistan and Afghanistan, and type O in East Africa, respectively [43-45]. The higher rate of FMDV evolution reported for the A-Iran 05 FMDV lineage in Afghanistan and Pakistan (1.2 × 10^−2^ nt/site/yr) [46] was similar to the molecular clock for the O CATHAY topotype estimated by this study. Therefore, genotypically and regionally variable evolutionary rates may in fact reflect real differences in the epidemiological dynamics and host-interaction of FMDV.

Although using a large database of FMDV isolates and generating a comprehensive picture of the O CATHAY topotype evolutionary history, this study has some limitations largely derived from the nature of the genetic data used for the analysis. The VP1 coding region, although defining only ~8% (639 nt of length) of the complete FMDV genome, is the most variable section of the FMDV genome and is historically used for tracing the movement and spread of FMD globally [1,4] and, furthermore, provides the basis for FMDV genotype definition [5]. Analysing a larger part of the FMDV genome, such as the whole capsid region or the full-length genome, would produce results with a higher resolution [47,48]. However, it should be noted that recombination events seem to be more widespread in other part of the genome [16,49,50], thus representing a limitation in interpreting results based on full-length genome analysis of large scale FMDV evolutionary studies. The ratio of per-site recombination to mutation rate here estimated from the full currently available FMDV type O CATHAY topotype VP1 coding sequences database is very low indicating that these results are not influenced by the process of recombination.

## Additional files

Additional file 1:
**FMDV type O CATHAY VP1 Philippines sequences database.** Designation and origin of the FMDV clinical samples (*n* = 112) collected from the Philippines between 1994 and 2005 and processed in this study. ^†^Date received by the WRLFMD was used where exact collection date was missing. Additional file 2:
**FMDV type O CATHAY VP1 sequences database.** Designation and origin of the VP1 sequences (*n* = 210) retrieved from either GenBank or the WRLFMD databases and belonging to the O CATHAY topotype. ^†^Date received by WRLFMD, year of collection or GenBank submission date were used where exact collection date was missing [51,52].
