# Supplementary material for: Phylodynamic reconstruction of O CATHAY topotype foot-and-mouth disease virus epidemics in the Philippines
Source: Vet Res. 2014 Aug 24;45(1):90. doi: 10.1186/s13567-014-0090-y (PMC4177241; doi:10.1186/s13567-014-0090-y)
Supplement: Additional file 1: — FMDV type O CATHAY VP1 Philippines sequences database. Designation and origin of the FMDV clinical samples (n = 112) collected from the Philippines between 1994 and 2005 and processed in this study. †Date received by the WRLFMD was used where exact collection date was missing. [file 13567_2014_90_MOESM1_ESM.docx]

| **Virus Designation** | **Tree Code** | **Region** | **Location** | **Date of Collection^†^** | **Species** | **GenBank No** | **Reference** |
| --- | --- | --- | --- | --- | --- | --- | --- |
| O/PHI/2/94 | O/PHI/2/94 | - | - | 06/12/1994^†^ | Porcine | KM243034 | This study |
| O/PHI/5/94 | O/PHI/5/94 | - | - | 06/12/1994^†^ | Porcine | KM243035 | This study |
| O/PHI/6/94 | O/PHI/6/94 | - | - | 06/12/1994^†^ | Porcine | KM243036 | This study |
| O/PHI/8/94 | O/PHI/8/94 | Calabarzon | Bagong Nayon | 08/09/1994 | Porcine | KM243037 | This study |
| O/PHI/10/94 | O/PHI/10/94 | Calabarzon | San Isidro | 28/10/1994 | Porcine | KM243038 | This study |
| O/PHI/11/94 | O/PHI/11/94 | Calabarzon | - | 07/12/1994 | Porcine | KM243039 | This study |
| O/PHI/12/94 | O/PHI/12/94 | Ilocos | - | 13/12/1994 | Porcine | KM243040 | This study |
| O/PHI/1/95 | O/PHI/1/95 | Central Luzon | Tenejero | 05/01/1995 | Porcine | KM243041 | This study |
| O/PHI/2/95 | O/PHI/2/95 | Ilocos | - | 12/01/1995 | Porcine | KM243042 | This study |
| O/PHI/3/95 | O/PHI/3/95 | Central Luzon | - | 15/01/1995 | Porcine | KM243043 | This study |
| O/PHI/5/95 | O/PHI/5/95 | Ilocos | - | 09/02/1995 | Porcine | DQ164946 | [8] |
| O/PHI/6/95 | O/PHI/6/95 | Central Luzon | Santa Ines | 23/03/1995 | Porcine | KM243044 | This study |
| O/PHI/9/95 | O/PHI/9/95 | NCR | - | 23/03/1995 | Porcine | KM243045 | This study |
| O/PHI/10/95 | O/PHI/10/95 | Bicol | - | 06/04/1995 | Porcine | KM243046 | This study |
| O/PHI/11/95 | O/PHI/11/95 | NCR | - | 06/04/1995 | Porcine | KM243047 | This study |
| O/PHI/12/95 | O/PHI/12/95 | Calabarzon | Calabuso | 03/10/1996^†^ | Porcine | KM243048 | This study |
| O/PHI/13/95 | O/PHI/13/95 | Calabarzon | Ampid | 03/10/1996^†^ | Porcine | KM243049 | This study |
| O/PHI/14/95 | O/PHI/14/95 | Central Luzon | - | 03/10/1996^†^ | Porcine | KM243050 | This study |
| O/PHI/1/96 | O/PHI/1/96 | Central Luzon | - | 03/10/1996^†^ | Porcine | KM243053 | This study |
| O/PHI/2/96 | O/PHI/2/96 | Central Luzon | - | 03/10/1996^†^ | Porcine | KM243054 | This study |
| O/PHI/3/96 | O/PHI/3/96 | Eastern Visayas | - | 03/10/1996^†^ | Porcine | KM243055 | This study |
| O/PHI/5/96 | O/PHI/5/96 | Bicol | - | 01/09/1996 | Porcine | KM243056 | This study |
| O/PHI/6/96 | O/PHI/6/96 | Ilocos | Aliaga | 01/10/1996 | Porcine | KM243057 | This study |
| O/PHI/7/96 | O/PHI/7/96 | Calabarzon | Mahabang Parang | 01/11/1996 | Porcine | KM243058 | This study |
| O/PHI/2/97 | O/PHI/2/97 | NCR | - | 16/01/1997 | Porcine | KM243059 | This study |
| O/PHI/3/97 | O/PHI/3/97 | Ilocos | - | 01/01/1997 | Porcine | KM243060 | This study |
| O/PHI/4/97 | O/PHI/4/97 | Central Luzon | Poblacion | 01/02/1997 | Porcine | KM243061 | This study |
| O/PHI/5/97 | O/PHI/5/97 | CAR | Guisad | 01/02/1997 | Porcine | KM243062 | This study |
| O/PHI/6/97 | O/PHI/6/97 | Central Luzon | - | 01/03/1997 | Porcine | KM243063 | This study |
| O/PHI/7/97 | O/PHI/7/97 | NCR | Payatas | 01/03/1997 | Porcine | KM243064 | This study |
| O/PHI/8/97 | O/PHI/8/97 | Central Luzon | Malibong Bata | 01/03/1997 | Porcine | KM243065 | This study |
| O/PHI/10/97 | O/PHI/10/97 | Bicol | Cabangan | 01/03/1997 | Porcine | KM243066 | This study |
| O/PHI/11/97 | O/PHI/11/97 | Bicol | - | 06/04/1997 | Porcine | KM243067 | This study |
| O/PHI/12/97 | O/PHI/12/97 | Central Luzon | - | 19/11/1997^†^ | Porcine | KM243070 | This study |
| O/PHI/13/97 | O/PHI/13/97 | Central Luzon | Balatong | 19/11/1997^†^ | Porcine | KM243071 | This study |
| O/PHI/14/97 | O/PHI/14/97 | Bicol | - | 19/11/1997^†^ | Porcine | KM243072 | This study |
| O/PHI/15/97 | O/PHI/15/97 | Central Luzon | Sampaga | 19/11/1997^†^ | Porcine | KM243073 | This study |
| O/PHI/16/97 | O/PHI/16/97 | Central Luzon | - | 19/11/1997^†^ | Porcine | KM243074 | This study |
| O/PHI/1/98 | O/PHI/1/98 | Central Luzon | - | 01/01/1998 | - | KM243075 | This study |
| O/PHI/2/98 | O/PHI/2/98 | Central Luzon | - | 01/01/1998 | - | KM243076 | This study |
| O/PHI/3/98 | O/PHI/3/98 | Central Luzon | Matatalaib | 01/01/1998 | - | KM243077 | This study |
| O/PHI/4/98 | O/PHI/4/98 | Central Luzon | - | 01/01/1998 | - | KM243078 | This study |
| O/PHI/5/98 | O/PHI/5/98 | Calabarzon | - | 01/01/1998 | - | KM243079 | This study |
| O/PHI/6/98 | O/PHI/6/98 | Calabarzon | - | 01/01/1998 | - | KM243080 | This study |
| O/PHI/8/98 | O/PHI/8/98 | Central Luzon | - | 01/01/1998 | - | KM243081 | This study |
| O/PHI/9/98 | O/PHI/9/98 | NCR | Nepomuceno | 01/01/1998 | - | KM243082 | This study |
| O/PHI/10/98 | O/PHI/10/98 | - | - | 01/01/1998 | - | KM243083 | This study |
| O/PHI/11/98 | O/PHI/11/98 | NCR | Malinta | 01/01/1998 | - | KM243084 | This study |
| O/PHI/12/98 | O/PHI/12/98 | - | - | 01/01/1998 | Porcine | KM243085 | This study |
| O/PHI/13/98 | O/PHI/13/98 | - | - | 01/01/1998 | Porcine | KM243086 | This study |
| O/PHI/14/98 | O/PHI/14/98 | - | - | 01/01/1998 | Porcine | KM243087 | This study |
| O/PHI/15/98 | O/PHI/15/98 | - | - | 01/01/1998 | Porcine | KM243088 | This study |
| O/PHI/16/98 | O/PHI/16/98 | - | - | 01/01/1998 | Buffalo | KM243089 | This study |
| O/PHI/18/98 | O/PHI/18/98 | - | - | 01/01/1998 | Porcine | KM243090 | This study |
| O/PHI/19/98 | O/PHI/19/98 | Central Luzon | - | 01/01/1998 | Porcine | KM243091 | This study |
| O/PHI/20/98 | O/PHI/20/98 | Central Luzon | - | 01/01/1998 | - | KM243092 | This study |
| O/PHI/21/98 | O/PHI/21/98 | Central Luzon | - | 01/01/1998 | Porcine | KM243093 | This study |
| O/PHI/22/98 | O/PHI/22/98 | Central Luzon | - | 01/01/1998 | Porcine | KM243094 | This study |
| O/PHI/23/98 | O/PHI/23/98 | Central Luzon | - | 01/01/1998 | Porcine | KM243095 | This study |
| O/PHI/25/98 | O/PHI/25/98 | NCR | Nepomuceno | 01/01/1998 | Porcine | KM243096 | This study |
| O/PHI/30/98 | O/PHI/30/98 | Calabarzon | - | 01/01/1998 | Porcine | KM243097 | This study |
| O/PHI/1/99 | O/PHI/1/99 | Central Luzon | - | 01/01/1999 | Porcine | KM243098 | This study |
| O/PHI/3/99 | O/PHI/3/99 | Central Luzon | Cupang West | 01/01/1999 | Porcine | KM243099 | This study |
| O/PHI/4/99 | O/PHI/4/99 | Central Luzon | Tungkong Mangga | 01/01/1999 | Porcine | KM243100 | This study |
| O/PHI/5/99 | O/PHI/5/99 | Western Visayas | - | 01/01/1999 | Porcine | KM243101 | This study |
| O/PHI/10/99 | O/PHI/10/99 | Central Luzon | - | 01/01/1999 | Porcine | KM243102 | This study |
| O/PHI/3/2000 | O/PHI/3/00 | Bicol | Tagas | 02/02/2000 | Porcine | KM243103 | This study |
| O/PHI/5/2000 | O/PHI/5/00 | Central Luzon | Santa Rosa | 08/02/2000 | Porcine | DQ164947 | [8] |
| O/PHI/6/2000 | O/PHI/6/00 | NCR | Fairview | 13/02/2000 | Porcine | KM243104 | This study |
| O/PHI/7/2000 | O/PHI/7/00 | Central Luzon | Santo Rosario | 21/02/2000 | Porcine | KM243105 | This study |
| O/PHI/8/2000 | O/PHI/8/00 | Central Luzon | Santa Cruz | 22/02/2000 | Porcine | KM243106 | This study |
| O/PHI/9/2000 | O/PHI/9/00 | Central Luzon | Pritil | 01/03/2000 | Porcine | KM243107 | This study |
| O/PHI/13/2000 | O/PHI/13/00 | Central Luzon | Santiago | 02/03/2000 | Porcine | DQ164948 | [8] |
| O/PHI/14/2000 | O/PHI/14/00 | Mimaropa | - | 11/03/2000 | Porcine | DQ164949 | [8] |
| O/PHI/15/2000 | O/PHI/15/00 | Mimaropa | - | 11/03/2000 | Porcine | KM243108 | This study |
| O/PHI/16/2000 | O/PHI/16/00 | Mimaropa | - | 11/03/2000 | Porcine | KM243109 | This study |
| O/PHI/17/2000 | O/PHI/17/00 | Mimaropa | - | 11/03/2000 | Porcine | KM243110 | This study |
| O/PHI/19/2000 | O/PHI/19/00 | Bicol | - | 07/04/2000 | Porcine | KM243111 | This study |
| O/PHI/23/2000 | O/PHI/23/00 | Calabarzon | San Andres | 24/05/2000 | Porcine | KM243112 | This study |
| O/PHI/24/2000 | O/PHI/24/00 | Bicol | Rawis | 24/05/2000 | Porcine | KM243113 | This study |
| O/PHI/26/2000 | O/PHI/26/00 | Central Luzon | Tabon | 29/06/2000 | Porcine | KM243114 | This study |
| O/PHI/27/2000 | O/PHI/27/00 | Central Luzon | - | 04/07/2000 | Porcine | KM243115 | This study |
| O/PHI/4/2001 | O/PHI/4/01 | Central Luzon | - | 01/01/2001 | Porcine | KM243116 | This study |
| O/PHI/5/2001 | O/PHI/5/01 | Central Luzon | - | 01/01/2001 | Porcine | KM243117 | This study |
| O/PHI/6/2001 | O/PHI/6/01 | Central Luzon | Dulong Bayan | 01/01/2001 | Porcine | KM243118 | This study |
| O/PHI/7/2001 | O/PHI/7/01 | Central Luzon | Dulong Bayan | 01/01/2001 | Porcine | KM243119 | This study |
| O/PHI/8/2001 | O/PHI/8/01 | Central Luzon | Poblacion | 01/01/2001 | Porcine | KM243120 | This study |
| O/PHI/9/2001 | O/PHI/9/01 | Central Luzon | Santo Cristo | 01/01/2001 | Porcine | KM243121 | This study |
| O/PHI/10/2001 | O/PHI/10/01 | Central Luzon | Partida | 01/01/2001 | Porcine | KM243122 | This study |
| O/PHI/5/2003 | O/PHI/5/03 | CAR | - | 10/02/2003 | Porcine | DQ164950 | [8] |
| O/PHI/10/2003 | O/PHI/10/03 | NCR | - | 04/03/2003 | Porcine | DQ164951 | [8] |
| O/PHI/14/2003 | O/PHI/14/03 | NCR | Dian | 21/03/2003 | Porcine | KM243123 | This study |
| O/PHI/17/2003 | O/PHI/17/03 | Central Luzon | Santa Filomena | 08/04/2003 | Porcine | DQ164952 | [8] |
| O/PHI/18/2003 | O/PHI/18/03 | Calabarzon | Balibago | 07/05/2003 | Porcine | KM243124 | This study |
| O/PHI/20/2003 | O/PHI/20/03 | Calabarzon | Balibago | 07/05/2003 | Porcine | KM243125 | This study |
| O/PHI/21/2003 | O/PHI/21/03 | Calabarzon | Pagrai | 13/05/2003 | Porcine | DQ164953 | [8] |
| O/PHI/23/2003 | O/PHI/23/03 | Calabarzon | - | 15/05/2003 | Porcine | DQ164954 | [8] |
| O/PHI/1/2004 | O/PHI/1/04 | Ilocos | Cabaroan Daya | 13/01/2004 | Porcine | DQ164955 | [8] |
| O/PHI/2/2004 | O/PHI/2/04 | Central Luzon | - | 16/01/2004 | Porcine | DQ164956 | [8] |
| O/PHI/3/2004 | O/PHI/3/04 | NCR | Kamuning | 05/02/2004 | Porcine | DQ164957 | [8] |
| O/PHI/4/2004 | O/PHI/4/04 | NCR | Nepomuceno | 24/03/2004 | Porcine | DQ164958 | [8] |
| O/PHI/5/2004 | O/PHI/5/04 | NCR | Pinagbuhatan | 01/06/2004 | Porcine | DQ164959 | [8] |
| O/PHI/6/2004 | O/PHI/6/04 | NCR | Dagat-Dagatan | 21/06/2004 | Porcine | DQ164960 | [8] |
| O/PHI/7/2004 | O/PHI/7/04 | Central Luzon | Ayson | 29/06/2004 | Porcine | DQ164961 | [8] |
| O/PHI/8/2004 | O/PHI/8/04 | Central Luzon | - | 14/07/2004 | Porcine | DQ164962 | [8] |
| O/PHI/9/2004 | O/PHI/9/04 | Calabarzon | Mayamot | 21/07/2004 | Porcine | DQ164963 | [8] |
| O/PHI/10/2004 | O/PHI/10/04 | NCR | Project 8 | 04/08/2004 | Porcine | DQ164964 | [8] |
| O/PHI/11/2004 | O/PHI/11/04 | NCR | - | 03/09/2004 | Porcine | DQ164965 | [8] |
| O/PHI/12/2004 | O/PHI/12/04 | Calabarzon | Calabuso | 29/09/2004 | Porcine | DQ164966 | [8] |
| O/PHI/1/2005 | O/PHI/1/05 | NCR | La Loma | 16/02/2005 | Porcine | KM243127 | This study |
| O/PHI/2/2005 | O/PHI/2/05 | NCR | - | 23/02/2005 | Porcine | KM243128 | This study |
| O/PHI/3/2005 | O/PHI/3/05 | NCR | La Loma | 03/03/2005 | Porcine | KM243131 | This study |
