# Supplementary material for: Phylodynamic reconstruction of O CATHAY topotype foot-and-mouth disease virus epidemics in the Philippines
Source: Vet Res. 2014 Aug 24;45(1):90. doi: 10.1186/s13567-014-0090-y (PMC4177241; doi:10.1186/s13567-014-0090-y)
Supplement: Additional file 2: — FMDV type O CATHAY VP1 sequences database. Designation and origin of the VP1 sequences (n = 210) retrieved from either GenBank or the WRLFMD databases and belonging to the O CATHAY topotype. †Date received by WRLFMD, year of collection or GenBank submission date were used where exact collection date was missing [51,52]. [file 13567_2014_90_MOESM2_ESM.docx]

| **Virus Designation** | **Tree Code** | **Country** | **Location** | **Date of Collection** | **Species** | **GenBank No** | **Reference** |
| --- | --- | --- | --- | --- | --- | --- | --- |
| O/CHA/Akesu/58 | O/CHA/Ake/58 | China | Akesu | 01/01/1958^†^ | Bovine | AJ131469 | Zhao et al., unpublished data |
| O/CHA/Akesu-MIII/58 | O/CHA/AkeOMIII/58 | China | Akesu | 01/01/1958^†^ | - | AY359854 | Wang et al., unpublished data |
| O/TAI/Ban/60 | O/TAI/Ban/60 | Thailand | Bangkok | 01/01/1960^†^ | Porcine | KM243030 | This study |
| O/HKN/21/70 | O/HKN/21/70 | Hong Kong | Hang Tau | 13/03/1970 | Porcine | AJ294911 | [6] |
| O/HKN/1/73 | O/HKN/01/73 | Hong Kong | Lantau Island | 01/01/1973 | Porcine | AJ294912 | [6] |
| O/HKN/19/73 | O/HKN/19/73 | Hong Kong | Ha Cheung Sha | 19/06/1973 | Bovine | AJ294913 | [6] |
| O/HKN/3/75 | O/HKN/03/75 | Hong Kong | Ping Shan | 23/12/1974 | Porcine | AJ294915 | [6] |
| O/HKN/27/77 | O/HKN/27/77 | Hong Kong | - | 01/01/1977 | Porcine | KM243031 | This study |
| O/HKN/33/77 | O/HKN/33/77 | Hong Kong | Tin Ping Shan | 01/01/1977 | Porcine | AJ294916 | [6] |
| O/AUR/Tha/81 | O/AUR/Tha/81 | Austria | Thalheim | 18/03/1981^†^ | Porcine | KM243032 | This study |
| O/HKN/14/82 | O/HKN/14/82 | Hong Kong | Hei Ling Chau | 25/02/1982 | Porcine | AJ294917 | [6] |
| O/GER/Wup/82 | O/GER/Wup/82 | Germany | Wuppertal | 16/06/1982^†^ | Porcine | KM243033 | This study |
| O/HKN/6/83 | O/HKN/06/83 | Hong Kong | Pok Fu Lam | 18/12/1982 | Bovine | AJ294919 | [6] |
| O/HKN/7/85 | O/HKN/07/85 | Hong Kong | Ma On Kong | 25/01/1985 | Porcine | AJ294920 | [6] |
| O/CHA/Gua/86 | O/CHA/Gua/86 | China | Guangdong | 01/01/1986^†^ | Porcine | AJ131468 | Zhao et al., unpublished data |
| O/HKN/12/91 | O/HKN/12/91 | Hong Kong | Shek Kwu Chau | 26/11/1991 | Porcine | AJ294921 | [6] |
| O/HKN/93 | O/HKN/93 | Hong Kong | - | 01/01/1993^†^ | Porcine | AJ131470 | Zhao et al., unpublished data |
| O/1685/RUS/95 | O/RUS/Mos/95 | Russia | Moscow | 16/06/1995 | Porcine | AJ004680 | Sherbakov et al., unpublished data |
| O/HKN/1/96 | O/HKN/01/96 | Hong Kong | Lau Fau Shan | 16/01/1996 | Porcine | KM243051 | This Study |
| O/HKN/7/96 | O/HKN/07/96 | Hong Kong | - | 06/02/1996 | Bovine | AJ294922 | [6] |
| O/HKN/16/96 | O/HKN/16/96 | Hong Kong | Lei Uk | 29/03/1996 | Porcine | KM243052 | This study |
| O/HKN/20/96 | O/HKN/20/96 | Hong Kong | - | 17/04/1996 | Bovine | AJ294924 | [6] |
| O/TAW/97 | O/TAW/97 | Taiwan | - | 01/04/1997^†^ | Porcine | AY593835 | [16] |
| O/TAW/Yun/97 | O/TAW/Yun/97 | Taiwan | Yunlin | 01/04/1997^†^ | Porcine | AF308157 | [13] |
| O/TAW/Chu/97 | O/TAW/Chu/97 | Taiwan | Chu-Pei | 01/04/1997^†^ | - | AF026168 | [14] |
| O/TAW/TL/97 | O/TAW/TL/97 | Taiwan | - | 01/04/1997^†^ | Porcine | AF030259 | Lai et al., unpublished data |
| O/TAW/Tao018/97 | O/TAW/Tao018/97 | Taiwan | Taoyuan | 01/04/1997^†^ | Porcine | AF095863 | [14] |
| O/TAW/Tai041/97 | O/TAW/Tai041/97 | Taiwan | Tainan | 01/04/1997^†^ | Porcine | AF095864 | [14] |
| O/TAW/Pin060/97 | O/TAW/Pin060/97 | Taiwan | Pingtun | 01/04/1997^†^ | Porcine | AF095865 | [14] |
| O/TAW/Tai077/97 | O/TAW/Tai077/97 | Taiwan | Taichung | 01/04/1997^†^ | Porcine | AF095866 | [14] |
| O/TAW/Hsi079/97 | O/TAW/Hsi079/97 | Taiwan | Hsinchu | 01/04/1997^†^ | Porcine | AF095867 | [14] |
| O/TAW/Nan089/97 | O/TAW/Nan089/97 | Taiwan | Nantou | 01/04/1997^†^ | Porcine | AF095868 | [14] |
| O/TAW/Tai109/97 | O/TAW/Tai109/97 | Taiwan | Taipei | 01/04/1997^†^ | Porcine | AF095869 | [14] |
| O/TAW/Tai110/97 | O/TAW/Tai110/97 | Taiwan | Taipei | 01/04/1997^†^ | Porcine | AF095870 | [14] |
| O/TAW/Tai111/97 | O/TAW/Tai111/97 | Taiwan | Taitung | 01/04/1997^†^ | Porcine | AF095871 | [14] |
| O/TAW/Tao113/97 | O/TAW/Tao113/97 | Taiwan | Taoyuan | 01/04/1997^†^ | Porcine | AF095872 | [14] |
| O/TAW/Hsi128/97 | O/TAW/Hsi128/97 | Taiwan | Hsinchu | 01/04/1997^†^ | Porcine | AF095873 | [14] |
| O/TAW/Yun136/97 | O/TAW/Yun136/97 | Taiwan | Yunlin | 01/04/1997^†^ | Porcine | AF095874 | [14] |
| O/TAW/Tai145/97 | O/TAW/Tai145/97 | Taiwan | Taipei | 01/04/1997^†^ | Porcine | AF095875 | [14] |
| O/TAW/Tai150/97 | O/TAW/Tai150/97 | Taiwan | Taipei | 01/04/1997^†^ | Porcine | AF095876 | [14] |
| O/TAW/Kao153/97 | O/TAW/Kao153/97 | Taiwan | Kaohsiung | 01/04/1997^†^ | Porcine | AF095877 | [14] |
| O/TAW/Chu158/97 | O/TAW/Chu158/97 | Taiwan | Chunhwa | 01/04/1997^†^ | Porcine | AF095879 | [14] |
| O/TAW/Mia165/97 | O/TAW/Mia165/97 | Taiwan | Miaoli | 01/04/1997^†^ | Porcine | AF095879 | [14] |
| O/TAW/Tai168/97 | O/TAW/Tai168/97 | Taiwan | Tainan | 01/04/1997^†^ | Porcine | AF095880 | [14] |
| O/TAW/Tai181/97 | O/TAW/Tai181/97 | Taiwan | Tainan | 01/04/1997^†^ | Porcine | AF095881 | [14] |
| O/TAW/Tai186/97 | O/TAW/Tai186/97 | Taiwan | Taichung | 01/04/1997^†^ | Porcine | AF095882 | [14] |
| O/TAW/Chu188/97 | O/TAW/Chu188/97 | Taiwan | Chunhwa | 01/04/1997^†^ | Porcine | AF095883 | [14] |
| O/TAW/Hsi189/97 | O/TAW/Hsi189/97 | Taiwan | Hsinchu | 01/04/1997^†^ | Porcine | AF095884 | [14] |
| O/TAW/Kao190/97 | O/TAW/Kao190/97 | Taiwan | Kaohsiung | 01/04/1997^†^ | Porcine | AF095885 | [14] |
| O/TAW/81/97 | O/TAW/81/97 | Taiwan | Yilan | 17/04/1997 | Porcine | KM243068 | This study |
| O/TAW/83/97 | O/TAW/83/97 | Taiwan | Taitung | 24/04/1997 | Porcine | KM243069 | This study |
| O/VIT/3/97 | O/VIT/03/97 | Vietnam | - | 26/08/1997^†^ | Porcine | AJ294930 | [6] |
| O-TW-185-97 | O/TAW/185/97 | Taiwan | - | 07/12/1997 | Porcine | GQ292726 | [51] |
| O-TW-205-98 | O/TAW/205/98 | Taiwan | Tainan | 07/01/1998 | Porcine | GQ292727 | [51] |
| O-TW-210-98 | O/TAW/210/98 | Taiwan | Yunlin | 23/01/1998 | Porcine | GQ292728 | [51] |
| O-TW-219-98 | O/TAW/219/98 | Taiwan | Tainan | 07/04/1998 | Porcine | GQ292729 | [51] |
| O/HKN/1/99 | O/HKN/01/99 | Hong Kong | Mong Tseng Tsuen | 05/01/1999 | Porcine | AJ294925 | [6] |
| O/TAW/4/99 | O/TAW/04/99 | Taiwan | Penghu | 01/02/1999 | Porcine | AJ294928 | [6] |
| O-TW-241-99 | O/TAW/241/99 | Taiwan | Yunlin | 14/02/1999 | Porcine | GQ292730 | [51] |
| O-TW-242-99 | O/TAW/242/99 | Taiwan | Yunlin | 20/02/1999 | Porcine | GQ292731 | [51] |
| O-TW-244-99 | O/TAW/244/99 | Taiwan | Penghu | 23/02/1999 | Porcine | GQ292732 | [51] |
| O/HKN/10/99 | O/HKN/10/99 | Hong Kong | Pak Sha Tsuen | 19/03/1999 | Porcine | AJ318836 | Knowles et al., unpublished data |
| O-TW-249-99 | O/TAW/249/99 | Taiwan | Pingtun | 15/04/1999 | Porcine | GQ292733 | [51] |
| O-TW-251-99 | O/TAW/251/99 | Taiwan | Kaohsiung | 20/04/1999 | Porcine | GQ292734 | [51] |
| O-TW-252-99 | O/TAW/252/99 | Taiwan | Tainan | 21/04/1999 | Porcine | GQ292735 | [51] |
| O-TW-253-99 | O/TAW/253/99 | Taiwan | Hsinchu | 29/04/1999 | Porcine | GQ292736 | [51] |
| O-TW-255-2000 | O/TAW/255/00 | Taiwan | Taoyuan | 22/10/2000 | Porcine | GQ292737 | [51] |
| O/CHA/YM/YN/2000 | O/CHA/YMYN/00 | China | Yunnan | 18/12/2000 | Porcine | HQ412603 | Xin et al., unpublished data |
| O/CHA/F29 | O/CHA/F29 | China | - | 01/01/2001^†^ | Porcine | AF403048 | Lou and Du, unpublished data |
| O/HKN/4/2001 | O/HKN/04/01 | Hong Kong | - | 01/01/2001 | Porcine | DQ164875 | [8] |
| O-TW-256-2001 | O/TAW/256/01 | Taiwan | Taipei | 25/02/2001 | Porcine | GQ292738 | [51] |
| O/HKN/S01/2001 | O/HKN/S01/01 | Hong Kong | - | 01/07/2001 | Porcine | JF968125 | [18] |
| O/HKN/S03/2001 | O/HKN/S03/01 | Hong Kong | - | 01/07/2001 | Porcine | JF968126 | [18] |
| O/HKN/S04/2001 | O/HKN/S04/01 | Hong Kong | - | 01/07/2001 | Porcine | JF968127 | [18] |
| O/HKN/S05/2001 | O/HKN/S05/01 | Hong Kong | - | 01/07/2001 | Porcine | JF968128 | [18] |
| O/HKN/19/2001 | O/HKN/19/01 | Hong Kong | Sheung Shui | 28/09/2001 | Porcine | DQ164876 | [8] |
| O/HKN/S06/2001 | O/HKN/S06/01 | Hong Kong | - | 01/10/2001 | Porcine | JF968129 | [18] |
| O/HKN/S09/2001 | O/HKN/S09/01 | Hong Kong | - | 01/10/2001 | Porcine | JF968130 | [18] |
| O/HKN/S10/2001 | O/HKN/S10/01 | Hong Kong | - | 01/10/2001 | Porcine | JF968131 | [18] |
| O/VIT/13/2002 | O/VIT/13/02 | Vietnam | - | 01/01/2002 | - | DQ165025 | [8] |
| O/HKN/S11/2002 | O/HKN/S11/02 | Hong Kong | - | 01/01/2002 | Porcine | JF968132 | [18] |
| O/HKN/S12/2002 | O/HKN/S12/02 | Hong Kong | - | 01/01/2002 | Porcine | JF968133 | [18] |
| O/HKN/S13/2002 | O/HKN/S13/02 | Hong Kong | - | 01/01/2002 | Porcine | JF968134 | [18] |
| O/HKN/S14/2002 | O/HKN/S14/02 | Hong Kong | - | 01/01/2002 | Porcine | JF968135 | [18] |
| O/HKN/1/2002 | O/HKN/01/02 | Hong Kong | Yuen Long | 22/01/2002 | Porcine | DQ164877 | [8] |
| O/HKN/3/2002 | O/HKN/03/02 | Hong Kong | Yuen Long | 31/01/2002 | Porcine | DQ164878 | [8] |
| O/HKN/2002 | O/HKN/02 | Hong Kong | - | 01/02/2002 | Porcine | AY317098 | [52] |
| O/HKN/S15/2002 | O/HKN/S15/02 | Hong Kong | - | 01/04/2002 | Porcine | JF968136 | [18] |
| O/HKN/S17/2002 | O/HKN/S17/02 | Hong Kong | - | 01/04/2002 | Porcine | JF968137 | [18] |
| O/HKN/S18/2002 | O/HKN/S18/02 | Hong Kong | - | 01/04/2002 | Porcine | JF968138 | [18] |
| O/HKN/S19/2002 | O/HKN/S19/02 | Hong Kong | - | 01/04/2002 | Porcine | JF968139 | [18] |
| O/HKN/S20/2002 | O/HKN/S20/02 | Hong Kong | - | 01/04/2002 | Porcine | JF968140 | [18] |
| O/HKN/S22/2002 | O/HKN/S22/02 | Hong Kong | - | 01/05/2002 | Porcine | JF968141 | [18] |
| O/HKN/S24/2002 | O/HKN/S24/02 | Hong Kong | - | 01/06/2002 | Porcine | JF968142 | [18] |
| O/HKN/S25/2002 | O/HKN/S25/02 | Hong Kong | - | 01/06/2002 | Porcine | JF968145 | [18] |
| O/HKN/S32/2002 | O/HKN/S32/02 | Hong Kong | - | 01/10/2002 | Porcine | JF968143 | [18] |
| O/HKN/S34/2002 | O/HKN/S34/02 | Hong Kong | - | 01/10/2002 | Porcine | JF968146 | [18] |
| O/HKN/S44/2002 | O/HKN/S44/02 | Hong Kong | - | 01/10/2002 | Porcine | JF968147 | [18] |
| O/HKN/S72/2003 | O/HKN/S72/03 | Hong Kong | - | 01/01/2003 | Porcine | JF968148 | [18] |
| O/HKN/S73/2003 | O/HKN/S73/03 | Hong Kong | - | 01/01/2003 | Porcine | JF968148 | [18] |
| O/HKN/S74/2003 | O/HKN/S74/03 | Hong Kong | - | 01/01/2003 | Porcine | JF968149 | [18] |
| O/HKN/S75/2003 | O/HKN/S75/03 | Hong Kong | - | 01/01/2003 | Porcine | JF968150 | [18] |
| O/HKN/S76/2003 | O/HKN/S76/03 | Hong Kong | - | 01/01/2003 | Porcine | JF968151 | [18] |
| O/HKN/2/2003 | O/HKN/02/03 | Hong Kong | - | 01/01/2003 | Porcine | DQ164879 | [8] |
| O/HKN/3/2003 | O/HKN/03/03 | Hong Kong | - | 01/01/2003 | Porcine | DQ164880 | [8] |
| O/HKN/S78/2003 | O/HKN/S78/03 | Hong Kong | - | 01/02/2003 | Porcine | JF968152 | [18] |
| O/HKN/S79/2003 | O/HKN/S79/03 | Hong Kong | - | 01/02/2003 | Porcine | JF968153 | [18] |
| O/HKN/S80/2003 | O/HKN/S80/03 | Hong Kong | - | 01/02/2003 | Porcine | JF968154 | [18] |
| O/HKN/S81/2003 | O/HKN/S81/03 | Hong Kong | - | 01/02/2003 | Porcine | JF968157 | [18] |
| O/HKN/S83/2003 | O/HKN/S83/03 | Hong Kong | - | 01/02/2003 | Porcine | JF968155 | [18] |
| O/HKN/S84/2003 | O/HKN/S84/03 | Hong Kong | - | 01/02/2003 | Porcine | JF968159 | [18] |
| O/CHA/XJ1/03 | O/CHA/XJ1/03 | China | - | 01/08/2003^†^ | Bovine | AY373583 | Li et al., unpublished data |
| O/HKN/3/2004 | O/HKN/03/04 | Hong Kong | - | 28/01/2004 | Porcine | DQ164881 | [8] |
| O/VIT/2/2004 | O/VIT/02/04 | Vietnam | Quang Nam | 01/02/2004 | Porcine | DQ165033 | [8] |
| O/HKN/4/2004 | O/HKN/04/04 | Hong Kong | - | 11/02/2004 | Porcine | DQ164882 | [8] |
| O/VIT/3/2004 | O/VIT/03/04 | Vietnam | Quang Nam | 01/03/2004 | Porcine | DQ165034 | [8] |
| O/HKN/6/2004 | O/HKN/06/04 | Hong Kong | - | 02/03/2004 | Porcine | DQ164883 | [8] |
| O/HKN/7/2004 | O/HKN/07/04 | Hong Kong | - | 18/03/2004 | Porcine | DQ164884 | [8] |
| O/HKN/0238/2004 | O/HKN/0238/04 | Hong Kong | - | 01/06/2004 | Porcine | JF968160 | [18] |
| O/HKN/1738/2004 | O/HKN/1738/04 | Hong Kong | - | 01/06/2004 | Porcine | JF968161 | [18] |
| O/HKN/2037/2004 | O/HKN/2037/04 | Hong Kong | - | 01/06/2004 | Porcine | JF968162 | [18] |
| O/HKN/2038/2004 | O/HKN/2038/04 | Hong Kong | - | 01/06/2004 | Porcine | JF968163 | [18] |
| O/HKN/2140/2004 | O/HKN/2140/04 | Hong Kong | - | 01/06/2004 | Porcine | JF968144 | [18] |
| O/HKN/2228/2004 | O/HKN/2228/04 | Hong Kong | - | 01/06/2004 | Porcine | JF968164 | [18] |
| O/HKN/2231/2004 | O/HKN/2231/04 | Hong Kong | - | 01/06/2004 | Porcine | JF968166 | [18] |
| O/HKN/2235/2004 | O/HKN/2235/04 | Hong Kong | - | 01/06/2004 | Porcine | JF968165 | [18] |
| O/HKN/2332/2004 | O/HKN/2332/04 | Hong Kong | - | 01/06/2004 | Porcine | JF968167 | [18] |
| O/HKN/2822/2004 | O/HKN/2822/04 | Hong Kong | - | 01/06/2004 | Porcine | JF968169 | [18] |
| O/HKN/2838/2004 | O/HKN/2838/04 | Hong Kong | - | 01/06/2004 | Porcine | JF968168 | [18] |
| O/HKN/3039/2004 | O/HKN/3039/04 | Hong Kong | - | 01/06/2004 | Porcine | JF968170 | [18] |
| O/HKN/S93/2004 | O/HKN/S93/04 | Hong Kong | - | 01/07/2004 | Porcine | JF968123 | [18] |
| O/HKN/S95/2004 | O/HKN/S95/04 | Hong Kong | - | 01/07/2004 | Porcine | JF968124 | [18] |
| O/HKN/S97/2004 | O/HKN/S97/04 | Hong Kong | - | 01/07/2004 | Porcine | JF968122 | [18] |
| O/HKN/8/2004 | O/HKN/08/04 | Hong Kong | - | 11/08/2004 | Porcine | DQ164885 | [8] |
| O/HKN/9/2004 | O/HKN/09/04 | Hong Kong | - | 11/08/2004 | Porcine | DQ164886 | [8] |
| O/HKN/10/2004 | O/HKN/10/04 | Hong Kong | - | 11/08/2004 | Porcine | DQ164887 | [8] |
| O/HKN/11/2004 | O/HKN/11/04 | Hong Kong | - | 11/08/2004 | Porcine | DQ164888 | [8] |
| O/HKN/12/2004 | O/HKN/12/04 | Hong Kong | - | 11/08/2004 | Porcine | DQ164889 | [8] |
| O/HKN/13/2004 | O/HKN/13/04 | Hong Kong | - | 21/12/2004 | Porcine | KM243126 | This study |
| O/HKN/P115/2005 | O/HKN/P115/05 | Hong Kong | - | 01/01/2005 | Porcine | JF968171 | [18] |
| O/HKN/P125/2005 | O/HKN/P125/05 | Hong Kong | - | 01/01/2005 | Porcine | JF968172 | [18] |
| O/VIT/1/2005 | O/VIT/01/05 | Vietnam | - | 01/01/2005 | Bovine | HQ116276 | [17] |
| O/HKN/9/2005 | O/HKN/09/05 | Hong Kong | - | 25/02/2005 | Porcine | KM243129 | This study |
| O/HKN/10/2005 | O/HKN/10/05 | Hong Kong | - | 25/02/2005 | Porcine | KM243130 | This study |
| O/HKN/P179/2005 | O/HKN/P179/05 | Hong Kong | - | 01/03/2005 | Porcine | JF968173 | [18] |
| O/HKN/12/2005 | O/HKN/12/05 | Hong Kong | - | 11/03/2005 | Porcine | KM243132 | This study |
| O/HKN/14/2005 | O/HKN/14/05 | Hong Kong | - | 14/03/2005 | Porcine | KM243133 | This study |
| O/HKN/15/2005 | O/HKN/15/05 | Hong Kong | - | 14/03/2005 | Porcine | KM243134 | This study |
| O/HKN/P235/2005 | O/HKN/P235/05 | Hong Kong | - | 01/05/2005 | Porcine | JF968158 | [18] |
| O/VIT/9/2005 | O/VIT/09/05 | Vietnam | Hai Duong | 30/05/2005 | Porcine | HQ116281 | [17] |
| O/VIT/11/2005 | O/VIT/11/05 | Vietnam | Ha Giang | 18/06/2005 | Porcine | HQ116282 | [17] |
| O/HKN/17/2005 | O/HKN/17/05 | Hong Kong | - | 04/07/2005 | Porcine | KM243135 | This study |
| O/HKN/18/2005 | O/HKN/18/05 | Hong Kong | - | 04/07/2005 | Porcine | KM243136 | This study |
| O/HKN/19/2005 | O/HKN/19/05 | Hong Kong | - | 04/07/2005 | Porcine | KM243137 | This study |
| O/HKN/20/2005 | O/HKN/20/05 | Hong Kong | - | 04/07/2005 | Porcine | KM243138 | This study |
| O/VIT/12/2005 | O/VIT/12/05 | Vietnam | Long An | 28/07/2005 | Porcine | KM243139 | This study |
| O/HKN/22/2005 | O/HKN/22/05 | Hong Kong | - | 15/11/2005 | Porcine | KM243140 | This study |
| O/HKN/23/2005 | O/HKN/23/05 | Hong Kong | - | 15/11/2005 | Porcine | KM243141 | This study |
| O/HKN/24/2005 | O/HKN/24/05 | Hong Kong | - | 21/11/2005 | Porcine | KM243142 | This study |
| O/HKN/25/2005 | O/HKN/25/05 | Hong Kong | - | 21/11/2005 | Porcine | KM243143 | This study |
| O/TAI/5/2005 | O/TAI/05/05 | Thailand | - | 26/11/2005 | Porcine | HQ116235 | [17] |
| O/TAI/6/2005 | O/TAI/06/05 | Thailand | - | 27/11/2005 | Porcine | HQ116236 | [17] |
| O/HKN/P370/2005 | O/HKN/P370/05 | Hong Kong | - | 01/12/2005 | Porcine | JF968174 | [18] |
| O/HKN/P371/2005 | O/HKN/P371/05 | Hong Kong | - | 01/12/2005 | Porcine | JF968175 | [18] |
| O/HKN/P372/2005 | O/HKN/P372/05 | Hong Kong | - | 01/12/2005 | Porcine | JF968176 | [18] |
| O/MAY/8/2005 | O/MAY/08/05 | Malaysia | Tanjong Sepat | 02/12/2005 | Porcine | HQ116202 | [17] |
| O/VIT/1/2006 | O/VIT/01/06 | Vietnam | Long An | 01/01/2006 | Porcine | HQ116284 | [17] |
| O/VIT/2/2006 | O/VIT/02/06 | Vietnam | Dong Thap | 11/01/2006 | Porcine | HQ116285 | [17] |
| O/VIT/3/2006 | O/VIT/03/06 | Vietnam | Tien Giang | 12/01/2006 | Porcine | HQ116286 | [17] |
| O/HKN/1/2006 | O/HKN/01/06 | Hong Kong | - | 26/01/2006 | Porcine | KM243144 | This study |
| O/HKN/2/2006 | O/HKN/02/06 | Hong Kong | - | 26/01/2006 | Porcine | KM243145 | This study |
| O/HKN/3/2006 | O/HKN/03/06 | Hong Kong | - | 26/01/2006 | Porcine | KM243146 | This study |
| O/HKN/4/2006 | O/HKN/04/06 | Hong Kong | - | 26/01/2006 | Porcine | KM243147 | This study |
| O/HKN/5/2006 | O/HKN/05/06 | Hong Kong | - | 26/01/2006 | Porcine | KM243148 | This study |
| O/HKN/6/2006 | O/HKN/06/06 | Hong Kong | - | 26/01/2006 | Porcine | KM243149 | This study |
| O/HKN/1/2007 | O/HKN/01/07 | Hong Kong | - | 10/01/2007 | Porcine | KM243150 | This study |
| O/HKN/P389/2007 | O/HKN/P389/07 | Hong Kong | - | 01/02/2007 | Porcine | JF968177 | [18] |
| O/HKN/P390/2007 | O/HKN/P390/07 | Hong Kong | - | 01/02/2007 | Porcine | JF968178 | [18] |
| O/HKN/P391/2007 | O/HKN/P391/07 | Hong Kong | - | 01/02/2007 | Porcine | JF968179 | [18] |
| O/HKN/P392/2007 | O/HKN/P392/07 | Hong Kong | - | 01/02/2007 | Porcine | JF968180 | [18] |
| O/HKN/P393/2007 | O/HKN/P393/07 | Hong Kong | - | 01/02/2007 | Porcine | JF968181 | [18] |
| O/HKN/2/2007 | O/HKN/02/07 | Hong Kong | - | 23/03/2007 | Porcine | KM243151 | This study |
| O/HKN/3/2007 | O/HKN/03/07 | Hong Kong | - | 25/10/2007 | Porcine | KM243152 | This study |
| O/HKN/4/2007 | O/HKN/04/07 | Hong Kong | - | 25/10/2007 | Porcine | KM243153 | This study |
| O/VIT/1/2008 | O/VIT/01/08 | Vietnam | Ho Chi Minh | 01/01/2008 | Porcine | HQ116291 | [17] |
| O/VIT/9/2008 | O/VIT/09/08 | Vietnam | Ho Chi Minh | 04/02/2008 | Porcine | KM243154 | This study |
| O/HKN/1/2008 | O/HKN/01/08 | Hong Kong | - | 10/11/2008 | Porcine | KM243155 | This study |
| O/HKN/2/2008 | O/HKN/02/08 | Hong Kong | - | 10/11/2008 | Porcine | KM243156 | This study |
| O/HKN/3/2008 | O/HKN/03/08 | Hong Kong | - | 10/11/2008 | Porcine | KM243157 | This study |
| O/HKN/4/2008 | O/HKN/04/08 | Hong Kong | - | 10/11/2008 | Porcine | KM243158 | This study |
| O/HKN/P395/2008 | O/HKN/P395/08 | Hong Kong | - | 01/12/2008 | Porcine | JF968182 | [18] |
| O/HKN/P397/2009 | O/HKN/P397/09 | Hong Kong | - | 01/01/2009 | Porcine | JF968183 | [18] |
| O/HKN/P398/2009 | O/HKN/P398/09 | Hong Kong | - | 01/01/2009 | Porcine | JF968184 | [18] |
| O/HKN/1/2009 | O/HKN/01/09 | Hong Kong | - | 04/01/2009 | Porcine | KM243159 | This study |
| O/HKN/2/2009 | O/HKN/02/09 | Hong Kong | - | 04/01/2009 | Porcine | KM243160 | This study |
| O/HKN/P399/2009 | O/HKN/P399/09 | Hong Kong | - | 01/02/2009 | Porcine | JF968185 | [18] |
| O/TAW/1/2009 | O/TAW/01/09 | Taiwan | Mai-Liao | 04/02/2009 | Porcine | KM243161 | This study |
| O-TW-257-2009 | O/TAW/257/09 | Taiwan | - | 17/02/2009 | Porcine | GQ292739 | [51] |
| O-TW-258-2009 | O/TAW/258/09 | Taiwan | - | 17/02/2009 | Porcine | GQ292740 | [51] |
| O/HKN/24/2010 | O/HKN/24/10 | Hong Kong | - | 06/12/2010 | Porcine | KM243162 | This study |
| O/HKN/25/2010 | O/HKN/25/10 | Hong Kong | - | 06/12/2010 | Porcine | KM243163 | This study |
| O/HKN/26/2010 | O/HKN/26/10 | Hong Kong | - | 06/12/2010 | Porcine | KM243164 | This study |
| O/HKN/3/2011 | O/HKN/03/11 | Hong Kong | - | 24/08/2011 | Porcine | KM243165 | This study |
| O/HKN/4/2011 | O/HKN/04/11 | Hong Kong | - | 24/08/2011 | Porcine | KM243166 | This study |
| O/HKN/5/2011 | O/HKN/05/11 | Hong Kong | - | 24/08/2011 | Porcine | KM243167 | This study |
| O/HKN/6/2011 | O/HKN/06/11 | Hong Kong | - | 24/08/2011 | Porcine | KM243168 | This study |
| O/HKN/7/2011 | O/HKN/07/11 | Hong Kong | - | 24/08/2011 | Porcine | KM243169 | This study |
| O/HKN/8/2011 | O/HKN/08/11 | Hong Kong | - | 14/11/2011 | Porcine | KM243170 | This study |
| O/HKN/9/2011 | O/HKN/09/11 | Hong Kong | - | 14/11/2011 | Porcine | KM243171 | This study |
| O/HKN/1/2013 | O/HKN/01/13 | Hong Kong | - | 02/04/2013 | Porcine | KM243172 | This study |
